# Lactobacilli Downregulate Transcription Factors in *Helicobacter pylori* That Affect Motility, Acid Tolerance and Antimicrobial Peptide Survival

**DOI:** 10.3390/ijms232415451

**Published:** 2022-12-07

**Authors:** Fanglei Zuo, Tanvi Somiah, Hanna G. Gebremariam, Ann-Beth Jonsson

**Affiliations:** Department of Molecular Biosciences, The Wenner-Gren Institute, Stockholm University, 10691 Stockholm, Sweden

**Keywords:** helicobacter, Lactobacillus, transcription factor, LL-37, motility

## Abstract

*Helicobacter pylori* infection triggers inflammation that may lead to gastritis, stomach ulcers and cancer. Probiotic bacteria, such as *Lactobacillus*, have been of interest as treatment options, however, little is known about the molecular mechanisms of *Lactobacillus*-mediated inhibition of *H. pylori* pathogenesis. In this work, we investigated the effect of *Lactobacillus* culture supernatants, so-called conditioned medium (CM), from two gastric isolates, *L. gasseri* and *L. oris*, on the expression of transcriptional regulators in *H. pylori*. Among the four known two-component systems (TCSs), i.e., ArsRS, FlgRS, CheAY and CrdRS, the flagellar regulator gene *flgR* and the acid resistance associated *arsS* gene were down-regulated by *L. gasseri* CM, whereas expression of the other TCS-genes remained unaffected. *L. gasseri* CM also reduced the motility of *H. pylori*, which is in line with reduced *flgR* expression. Furthermore, among six transcription factors of *H. pylori* only the ferric uptake regulator gene *fur* was regulated by *L. gasseri* CM. Deletion of *fur* further led to dramatically increased sensitivity to the antimicrobial peptide LL-37. Taken together, the results highlight that released/secreted factors of some lactobacilli, but not all, downregulate transcriptional regulators involved in motility, acid tolerance and LL-37 sensitivity of *H. pylori*.

## 1. Introduction

*Helicobacter pylori* is a Gram-negative bacterium that colonizes the stomach of approximately half of the human population worldwide. Infection by *H. pylori* can trigger the development of gastroduodenal ulcers, mucosa-associated lymphoid tissue lymphoma, and gastric carcinoma [1]. *H. pylori* is able to persistently colonize the hostile environment of the human stomach by expressing virulence factors, such as flagella, cytotoxins and adhesins [2]. However, *H. pylori* possesses a small genome of approximately 1.6 Mb and encodes only a few known transcriptional regulators, which control the expression of genes involved in bacterial metabolism and pathogenicity [3]. Among 14 bona fide transcription factors, there are four two-component systems (ArsRS for acid acclimation, FlgRS and CheAY for flagellar motility, and CrdRS for copper resistance); two transcriptional regulators HspR and HrcA involved in the stress response; two transcriptional regulators Fur and NikR involved in metal ion homeostasis; and two orphan response regulators HP1043 and HP1021 with unknown or stress-related functions [4].

At the site of colonization, the bacteria face the innate immune system. Antimicrobial peptides (AMPs) are produced in response to bacterial infection and can be found in considerable amounts at various locations in the human body [5]. LL-37 is a human cathelicidin that is secreted from epithelial cells and released from neutrophils [6]. Human beta defensin-2 (hBD2) is a defensin that is produced from epithelial cells and at the lesional skin [7,8]. *H. pylori* is sensitive to both of these AMPs in vitro and can also upregulate hBD2 and LL-37 in gastric cells [9,10]. There is evidence that *H. pylori* can evade the killing effects of AMPs in the gastric tract [11].

*Lactobacillus* is a commensal group of bacteria that colonizes the human stomach and has the ability to exclude bacterial pathogens and prevent infections by direct and indirect mechanisms known as colonization resistance [12]. Some of these lactobacilli are used as an alternative or adjunct therapy to antibiotics for the eradication of *H. pylori,* even though the underlying mechanisms of the antagonistic effects are still incompletely understood [13]. Previous studies have suggested that lactobacilli can interfere with *H. pylori* pathogenesis by affecting virulence gene expression. For example, the bacteriocin reuterin from a *L. reuteri* strain has been reported to inhibit the transcription levels of *vacA* and *flaA* in *H. pylori* [14]. It has also been reported that certain lactobacilli strains can influence the transcription levels of the adhesin gene *sabA* of *H. pylori* [15].

In this work, we investigated the effect of lactobacilli supernatants, so-called conditioned medium (CM), on the four two-component systems of *H. pylori* and found that *L. gasseri* downregulated the *flgR* regulator of motility and the acid resistance gene *arsS*, whereas *L. oris* did not affect any of the regulator genes tested. Motility was impaired by incubation in *L. gasseri* supernatant, confirming an effect on the corresponding phenotype. Among the six additional transcriptional regulators tested, only *fur*, which is necessary for colonization and ferric regulation, was downregulated by *L. gasseri* supernatant. Deletion of *fur* led to increased sensitivity to LL-37. Taken together, the data show that certain lactobacilli strains can affect the motility, acid susceptibility and antimicrobial peptide resistance of *H. pylori* by downregulating key regulatory genes.

## 2. Results

### 2.1. Lactobacilli Affect the Gene Expression of flgR and arsS in H. pylori

**Figure 1:**
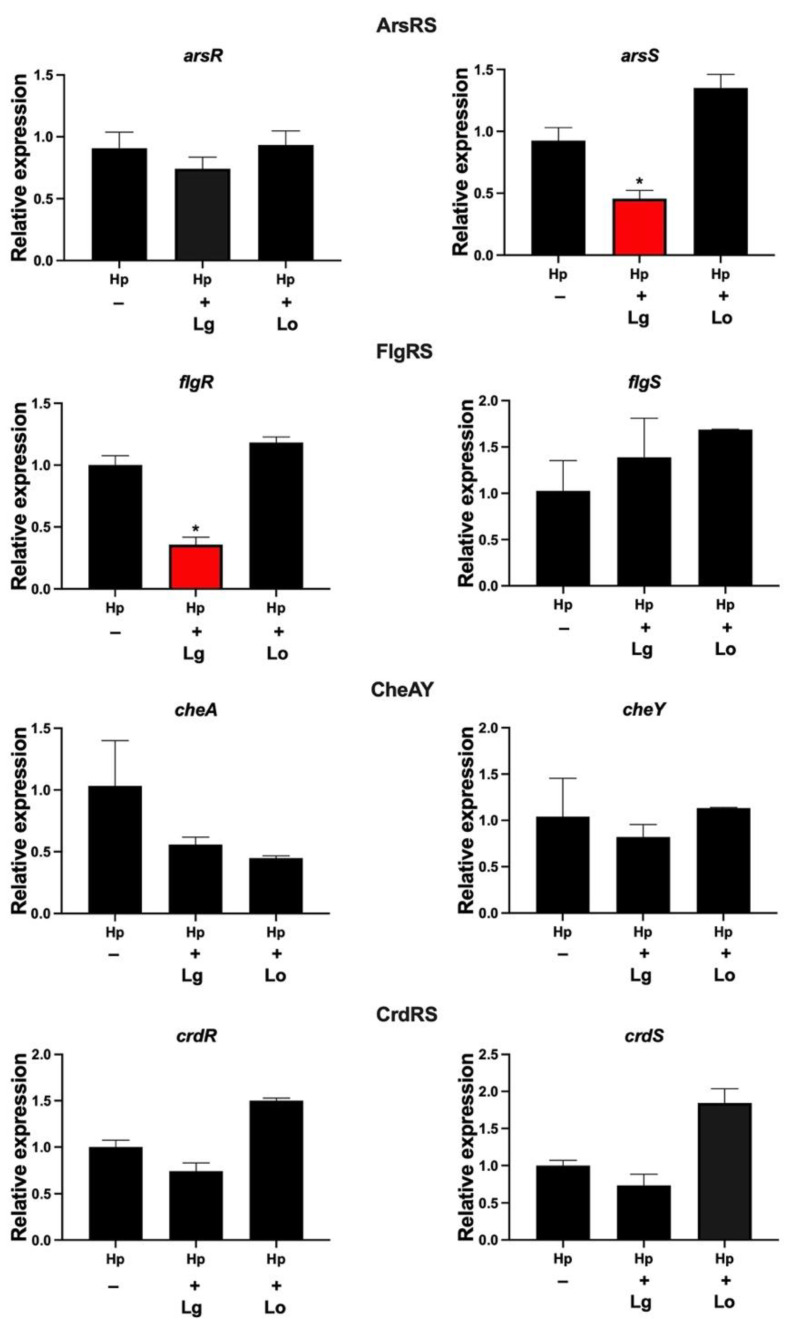
*L. gasseri* CM affects the gene expression of *arsS* and *flgA.* Gene expression of *H. pylori* (Hp) two-component systems analyzed by qPCR after incubation for 2 h in CM from *L. gasseri* or *L. oris* (Lo). Target mRNA levels were normalized to those of the housekeeping gene *gyrB*. Gene expression levels in the control were set to 1. Data are presented as the means and standard deviation of duplicate samples and are average of three independent experiments. * *p* < 0.05.

Two-component systems (TCSs) are important for the regulation of various bacterial factors. To assess the effect of lactobacilli on the expression of TCSs in *H. pylori*, we used supernatants, so-called conditioned medium (CM), from lactobacilli. *H. pylori* incubated with CM for 2 h was examined by qPCR for the expression of the genes of the four known TCSs, i.e., ArsRS, FlgRS, CheAY and CrdRS. Most genes were unaffected by incubation in CM, but two genes were significantly downregulated by lactobacilli. *H. pylori* incubated in *L. gasseri* CM showed a significant reduction in the expression of *flgR*, which is involved in flagella regulation, and in the expression of *arsS,* which is involved in acid adaptation (Figure 1). There were no significant changes in the expression of these genes when *H. pylori* was coincubated with *L. oris* CM. Thus, certain lactobacilli can affect the expression of genes encoding *H. pylori* TCSs. In summary, a soluble or secreted component(s) of *L. gasseri*, but not *L. oris*, both of which were isolated from gastric biopsies, downregulated the response regulator gene *flgR*, which is involved in motility, and the sensory histidine kinase gene *arsS*, which is involved in acid resistance.

### 2.2. Lactobacilli Affect H. pylori Motility in a Strain-Specific Manner

**Figure 2:**
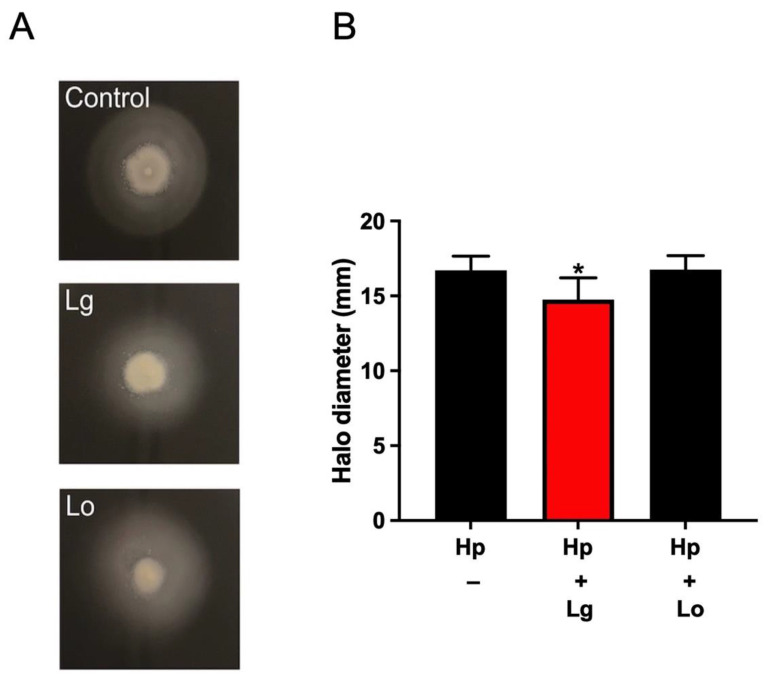
*L. gasseri* CM reduces *H. pylori* motility. The effect of lactobacilli CM on the motility of *H. pylori*. (**A**) Bacteria were stab inoculated with a pipette tip in Brucella motility agar and incubated at 37 °C under microaerobic conditions for 5 days. A representative agar plate is shown. (**B**) The halo diameter was measured in three independent experiments. Data are presented the means and standard deviation of duplicate samples and are average of three independent experiments. * *p* < 0.05.

**Figure 3:**
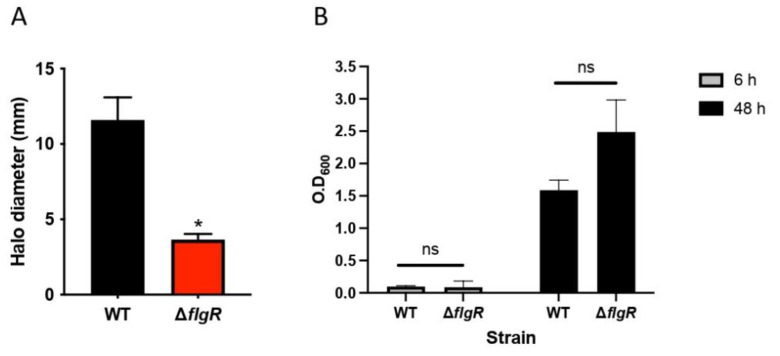
The motility phenotypes of wild-type *H. pylori* and the ∆*flgR* mutant. (**A**) The motility of *H. pylori* and mutant strains in semisolid Brucella motility agar. Bacteria were inoculated into Brucella motility agar and incubated at 37 °C under microaerobic conditions for 5 days. The growth halo diameters were measured and are presented as the mean ± SD from measurements of five independent experiments, * *p* < 0.05, ns: non-significant. (**B**) Growth of *H. pylori* and the ∆*flgR* mutant in medium BB10 over time was measured by OD_600nm_ at 6 h and 48 h of incubation. Data are represented as the mean ± standard deviation from three independent experiments.

Motility is an important virulence factor of *H. pylori.* It is well known that the two-component system FlgRS is involved in motility and flagellar gene regulation in *H. pylori* [16]. Since *flgR* was downregulated by *L. gasseri*, we next assessed whether lactobacilli strains might affect *H. pylori* motility. To examine the effect of lactobacilli CM on the motility of *H. pylori*, we inoculated the *H. pylori* wild-type strain in motility agar supplemented with CM of lactobacilli. As shown in Figure 2, the motility halo diameter of *H. pylori* in motility agar supplemented with CM of *L. gasseri* was slightly, but significantly, decreased compared with that of the control. However, the motility halo diameter in motility agar supplemented with CM of *L. oris* remained unaffected. To confirm the function of *flgR* in the motility of the *H. pylori* strain, we assessed the motility of a deletion mutant ∆*flgR* strain and the wild-type strain. As expected, the *H. pylori* wild-type strain was fully motile in motility agar, while the Δ*flgR* mutant strain showed impaired motility (Figure 3A). The Δ*flgR* mutant strain showed a colony phenotype and growth rate similar to those of the wild-type strain (Figure 3B). These results suggest that *L. gasseri*, but not *L. oris*, can inhibit the motile capacity of *H. pylori*, possibly by repressing the expression of genes associated with motility functions, such as *flgR*.

### 2.3. The ArsRS System Influences the Acid Sensitivity of H. pylori at pH 2

**Figure 4:**
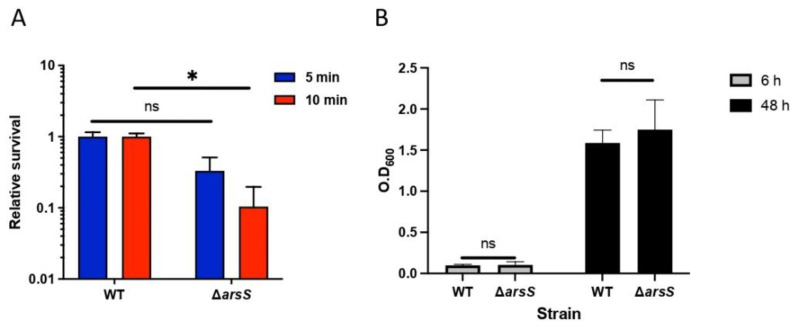
The effect of pH on the survival of *H. pylori* wild-type and ∆*arsS* mutant. (**A**) Bacteria were incubated in pH 2.0 for 5 min or 10 min. Viable counts were determined by plating. Survival is expressed relative to the untreated control. Data are presented as the means and standard deviation of duplicate samples and are representative of three independent experiments. * *p* < 0.05, ns: non-significant. (**B**) Growth of *H. pylori* and the ∆arsS mutant in medium BB10 over time was measured by OD_600nm_ at 6 h and 48 h of incubation. Data are represented as the mean ± standard deviation from three independent experiments.

The ability to survive in an acidic environment is an important survival factor of *H. pylori.* The ArsRS system is involved in acid adaptation [17]. Since *arsS* was downregulated by *L. gasseri*, we assessed the sensitivity of *H. pylori* to environmental stress by incubating *H. pylori* cells at pH 2 for 5 or 10 min. For this experiment, we constructed an *H. pylori* ∆*arsS* mutant strain, which showed a colony phenotype and growth similar to those of the wild-type strain (Figure 4A), to confirm the function of *arsS* in acid adaptation [18]. As expected, the Δ*arsS* mutant strain was significantly more sensitive to pH 2 than the *H. pylori* wild-type strain (Figure 4B), which is in line with data reported for other strains that show the dependence of *H. pylori* on the ArsRS system to express proteins required for acid acclimation, for example in strains 26695 and 43504 ArsRS regulates acid-induced expression of α-carbonic anhydrase [19].

### 2.4. Lactobacillus Gasseri Downregulates the Ferric Uptake Regulator

**Figure 5:**
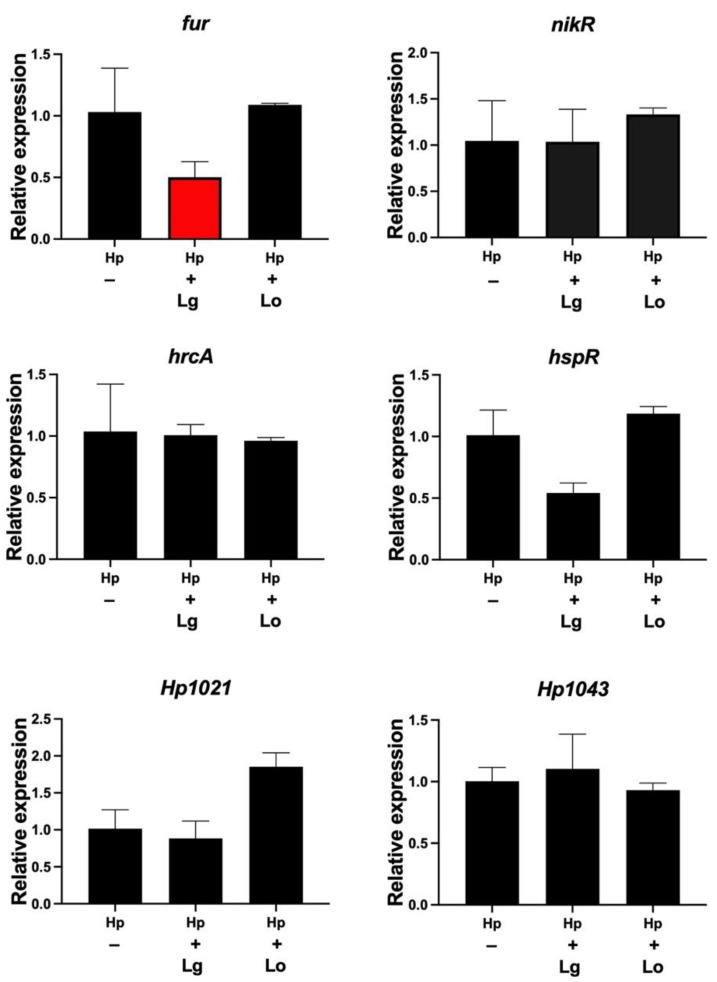
*L. gasseri* CM affects fur gene expression. Gene expression of *H. pylori* (Hp) transcription factors analyzed by qPCR after incubation for 2 h in CM from *L. gasseri* or *L. oris* (Lo). Target mRNA levels were normalized to those of the housekeeping gene *gyrB*. Gene expression levels in the control were set to 1. Data are presented as the means and standard deviation of duplicate samples and are average of three independent experiments.

We next investigated the expression of six transcription factors, i.e., *fur*, *nikR*, *hspR*, *hrcA*, *Hp1021* and *Hp1043*, by qPCR after incubation of *H. pylori* in lactobacilli conditioned medium (CM). *H. pylori* incubated for 2 h in *L. gasseri* CM showed a significant reduction in *fur*, which is involved in ferric uptake and colonization. There was no significant change in the expression of *fur* when *H. pylori* was coincubated with *L. oris* CM. The expression levels of genes for nickel homeostasis (*nikR*), heat shock (*hspR*, *hrcA*) and orphan regulators (*Hp1021*, *Hp1043*) did not significantly change in response to CM from any of the *Lactobacillus* strains (Figure 5). These data demonstrate that *L. gasseri*, but not *L. oris*, reduced the gene expression of the transcriptional global regulator *fur*.

### 2.5. Fur Is Involved in Antimicrobial Peptide LL-37 Resistance in H. pylori

**Figure 6:**
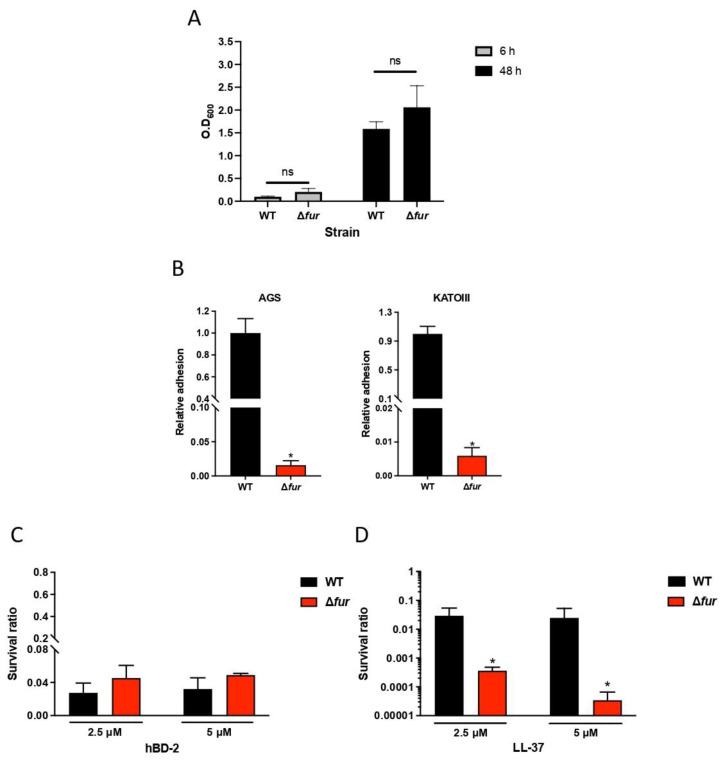
Survival of *H. pylori* and ∆*fur* in the presence of antimicrobial peptides. (**A**) Growth of *H. pylori* WT and the ∆*fur* mutant in medium BB10 over time was measured by OD_600nm_ at 6 h and 48 h of incubation. Data are represented as the mean ± standard deviation from three independent experiments. (**B**) Attachment of *H. pylori* wild-type and the ∆*fur* mutant to human epithelial gastric cells. Attachment to the gastric epithelial cell lines AGS (left panel) and KATO-III (right panel) of *H. pylori* at an MOI of 100 for 2 h. Unbound bacteria were removed by washing, and the adherent *H. pylori* were determined by serial dilution and plating. Data represent the relative adhesion ratio to the wild-type from three independent experiments with duplicate samples. The error bars represent the standard error of the mean. * *p* < 0.05, ns: non-significant. (**C**) The *H. pylori* wild-type and ∆*fur* mutant strains were incubated with hBD2 (2.5 or 5 μM) or (**D**) LL-37 (2.5 or 5 μM) for 2 h at 37 °C. Viable counts were determined by serial dilution and plating. The survival ratio is expressed relative to the untreated control. Data are presented as the means ± SD from three independent experiments. * *p* < 0.05.

Fur has been extensively studied by gene expression analysis and has been linked to reduced colonization [20]. Epithelial cells are known to secrete antimicrobial peptides, such as hBD-2 and LL-37, in response to *H. pylori* infection [9,21]. Since *fur* is important for colonization, we next investigated whether *fur* might influence the ability of bacteria to survive antimicrobial peptides (AMPs). To test this hypothesis, we assessed the survival of the wild-type and Δ*fur* strains in the presence of two AMPs, LL-37 and hBD-2. A Δ*fur* mutant was constructed in strain 67:21 and showed a colony phenotype and growth similar to those of the wild-type strain (Figure 6A), but it lost the ability to attach to host cells (Figure 6B), as previously shown for strain 7.13 [22]. The hBD-2 peptide showed a strong bactericidal effect against *H. pylori*, with no difference between the wild-type and ∆*fur* mutant strains and no significant difference between 2.5 µM and 5 µM hBD-2 (Figure 6C). The bactericidal effect of LL-37 was strong against wild-type *H. pylori;* however, the ∆*fur* mutant was extremely sensitive to LL-37, with 100-fold and 1000-fold reductions in viability after incubation with 2.5 and 5 μM LL-37, respectively, compared to wild-type *H. pylori* (Figure 6D). These results suggest that the expression of Fur plays an important role in *H. pylori* resistance to antimicrobial peptide LL-37.

## 3. Discussion

In this study, we explored the gene expression of two-component systems and transcription factors of *H. pylori* after incubation with lactobacilli. The expression levels of the motility-related gene *flgR* and the acid resistance-associated gene *arsS* were reduced after treatment with conditioned medium (CM) from *L. gasseri* but not after treatment with CM from *L. oris*. Among the tested transcription factors, only the ferric uptake-associated transcription factor *fur* was regulated by lactobacilli, and it was found to dramatically affect the resistance of *H. pylori* to the antimicrobial peptide LL-37.

It is well known that the two-component system FlgRS is involved in motility and flagellar gene regulation in *H. pylori* [16]. Numerous factors can influence the establishment of *H. pylori* colonization either directly or indirectly. One of these factors is the ability of *H. pylori* to be fully motile. Chemotaxis and helical rod shapes promote flagellar motility away from the acidic lumen to the preferred niche of *H. pylori*, which is on and close to gastric epithelial cells [23]. Incubation of *H. pylori* in *L. gasseri* CM downregulated the expression of the motility-related regulator *flgR*. Furthermore, when *H. pylori* was incubated with *L. gasseri* CM, motility was slightly but significantly reduced. It is tempting to speculate that the downregulation of *flgR* expression strongly contributes to the observed reduction in motility; however, we can not exclude the possibility that the regulation of other factors not tested also influenced motility. In other pathogens, such as *Escherichia coli*, *Pseudomonas aeruginosa*, *Clostridium difficile*, and *Yersinia enterocolitica*, flagellin and/or the distally located flagellar cap protein have been reported to function as adhesins [24,25]. However, there is no evidence of specific attachment of *H. pylori* flagella to epithelial cells, although it has been speculated that flagella have a role in cell adhesion [26]. During initial infection of the stomach lumen, urease-dependent ammonia production locally raises the pH, which promotes bacterial survival. We found that *L. gasseri* CM decreased the expression of *arsS*, which affects the expression of numerous genes, including urease genes [17]. It is reasonable to assume that the influence of secreted or released factors from *L. gasseri* on *H. pylori* regulatory genes modifies the virulence properties of the pathogen.

Among the tested transcriptional regulators, only the ferric uptake regulator *fur* was regulated by *L. gasseri*. Fur is a global regulator known to interact with the apo- and/or the holo-regulator on more than 200 identified target gene loci involved in metal homeostasis, acidic response, and virulence [27]. Fur has also been shown to regulate the transcription of several genes that may be important for cell adhesion [27], and Fur seems to directly or indirectly regulate other unknown factors that are essential for *H. pylori* adhesion to gastric epithelial cells. Analysis of the adhesion properties of the wild-type and Δ*fur* mutant indicated that Fur plays a critical role in *H. pylori* attachment to host epithelial cells. Even though many Fur-regulated genes have been shown to play a role in colonization [20], none of them have been previously shown to be directly involved in host cell attachment. The exact mechanism of the adherence deficiency of the *H. pylori* 67:21 Δ*fur* mutant needs to be further determined, with emphasis on the downstream adhesion-related genes that are positively regulated by Fur. Furthermore, the reduced adhesion ability and the high sensitivity to antimicrobial peptide LL-37 of the Δ*fur* mutant may be a reason for its deficiency in colonization of the mouse stomach [20]. The physiological concentration of LL-37 ranges between 0.03 μg/mL in human plasma and 8 μg/mL in bronchoalveolar lavage fluid, and it can reach up to 7 mg/mL in psoriatic lesions of inflammatory conditions [7,8,28]. Surface gastric epithelial cells and epithelial cells in the fundic glands of patients with gastritis can constitutively produce LL-37, and its production is upregulated by *H. pylori* infection [9].

*Helicobacter pylori* is the main risk factor for developing stomach cancer. Today, treatment with antibiotics that cure a majority of infections is recommended [29]. However, the use of antibiotics against *H. pylori* infection causes side effects and in particular leads to increased resistance of *H. pylori* as well as other pathogens to antibiotics. Indeed, the antimicrobial eradication rate of *H. pylori* has been declining globally because of antibiotic resistance [30,31]. A promising alternative or adjunct therapy is probiotic administration, which is generally prescribed as a supplement to standard therapy to reduce the adverse effects caused by antibiotics [32,33]. In particular, probiotics, such as lactobacilli, can combat *H. pylori* through multiple strategies; however, the molecular mechanisms underlying these antagonistic effects are still largely unknown [13,34,35]. In this study, since we see that the effect of lactobacilli on *H. pylori* is through a secreted component that does not require the two bacteria to be in contact with each other, it is tempting to speculate that the active molecule or molecules could be directly affecting virulence factors, as seen in the effect of *L. rhamnosus* on the Shiga toxin 2 mRNA expression in *E. coli,* without affecting viability [36]. Furthermore, motility of *S. typhimurium* is impaired by the secretion of a small, heat resistance compound(s) by *L. acidophilus* that works by depolarizing their flagellar motor, which is also a possible mechanism by which motility is impaired in the presence of *L. gasseri* in this study [34].

In summary, in this work, we demonstrated that lactobacilli supernatants can reduce the expression of two component systems affecting flagellar motility and pH sensitivity. Furthermore, the expression of *fur*, a global regulator controlling ferric uptake and colonization, was reduced by lactobacilli and was found to be important for resistance to the antimicrobial peptide LL-37, which is present at mucosal surfaces. In summary, we conclude that certain lactobacilli alter the expression of transcriptional regulators of *H. pylori* that may affect motility, LL-37 resistance and acid acclimatization.

## 4. Materials and Methods

### 4.1. Bacterial Strains, Media and Growth Conditions

The bacterial strains used in this study are listed in Table 1. The *H. pylori* strain 67:21 [37] was grown on Columbia blood agar plates (Acumedia, San Bernardino, CA, USA) supplemented with 8% defibrinated horse blood and 8% inactivated horse serum (Håtunalab, Stockholm, Sweden) (CBA) or in Brucella broth supplemented with 10% heat-inactivated fetal bovine serum (FBS) (BB10) at 37 °C under microaerophilic conditions, i.e., in an incubator with 5% O_2_, 10% CO_2_ and 85% N_2_. Lactobacilli were grown on Rogosa agar plates and cultured overnight in MRS broth (Oxoid, Göteborg, Sweden) at 37 °C and 5% CO_2_ in a humidified environment. *Escherichia coli* strains were routinely grown in LB medium at 37 °C. For the selection of *H. pylori* mutants, 5 μg/mL chloramphenicol was used. For the selection of plasmid-bearing *E. coli*, 10 μg/mL chloramphenicol was used.

### 4.2. Preparation of Conditioned Medium

Conditioned medium (CM) from lactobacilli was prepared by incubating lactobacilli in RPMI 1640 at 2 × 10^7^ CFU/mL at 37 °C and 5% CO_2_ for 2 h. The suspension was filtered through a 0.2-μM sterile filter to remove the bacterial cells [15].

### 4.3. qPCR Analysis

Incubation of *H. pylori* 67:21 wild-type and mutants in lactobacilli CM was performed for 2 h, based on changes in gene expression in this strain of *Heliocbacter pylori* 67:21 at a 2 h time point that have been previously studied [15]. These samples were then resuspended in lysis buffer (30 mM Tris-HCl, 1 mM EDTA, 30 mg/mL lysozyme, and proteinase K; pH 8.0) and incubated for 20 min at room temperature, with 10 s of vortexing and 2 min rest cycles. RNA was isolated using an RNeasy kit (Qiagen, Stockholm, Sweden) according to the manufacturer’s instructions. SuperScript VILO Mastermix (Thermo Fisher Scientific, Uppsala, Sweden) was used to synthesize the cDNA. Quantitative PCR (qPCR) was performed using a LightCycler 480 (Roche, Solna, Sweden) and a SYBR Green I Master kit (Roche, Sweden). The primers used are listed in Table 2. The PCR program was as follows: initial denaturation at 95 °C for 10 min followed by amplification for 40 cycles with denaturation at 95 °C for 10 s, annealing at 54 °C for 20 s, and extension at 72 °C for 20 s. The expression was analyzed by the 2(-ΔΔCt) method and was normalized to that of the housekeeping gene *gyrB* encoding DNA gyrase B. The expression levels were given as the fold change relative to the control samples.

### 4.4. Construction of H. pylori Deletion Mutants

The donor DNA constructs were achieved by fusing the upstream DNA of the target gene (amplified from the genome of *H. pylori* 67:21 by the up primer pair), the chloramphenicol resistance cassette (amplified from the plasmid pACYC184 by the Cmr primer pair), and the downstream DNA of the target gene (amplified by the down primer pair) by overlap PCR. The fusion products were inserted into the pJET1.2/blunt cloning vector (Thermo Fisher Scientific, Sweden), and the ligation mixtures were used to transform *E. coli* DH5α (Invitrogen). High-fidelity Phusion DNA polymerase (Thermo Fisher Scientific) was used for all PCRs. The *H. pylori* 67:21 mutants were created by electroporation of the *H. pylori* 67:21 wild-type with these plasmids and selection for chloramphenicol resistance as described previously [38]. The desired mutants with target genes replaced by an antibiotic resistance cassette were confirmed by PCR and sequencing (Eurofins, Hamburg, Germany).

### 4.5. Motility Assay

Soft-agar assays were performed using Brucella broth, 2.5% (*v*/*v*) FBS, and 0.35% Bacto agar as previously described [39]. To test the effect of lactobacilli on the motility of *H. pylori* 67:21, 5 mL CM from lactobacilli was mixed with 15 mL soft agar as described above. *H. pylori* 67:21 strains were stab inoculated with a pipette tip into soft agar plates and incubated for 5 days under microaerophilic conditions, and then the growth halo diameters were measured. Experiments using soft agar with 5 ml added CM compared to soft agar without added CM, resulted as expected in slightly larger halo diameters, since agar with CM had been diluted to give a lower agar concentration. These experiments were never compared to each other. 

### 4.6. Antimicrobial Peptide Susceptibility

To determine the sensitivity of the *H. pylori* 67:21 wild-type and Δ*fur* mutant strains to synthetic antimicrobial peptide human beta defensin 2 (hBD-2) and LL-37 (Innovagen), bacterial strains were suspended in RPMI 1640 at ~2 × 10^7^ CFU/mL, and the antimicrobial peptides were added at final concentrations of 2.5 and 5 mM, respectively. After incubation for 2 h at 37 °C, the bacterial suspensions were plated on CBA and incubated at 37 °C microaerobically for 5 days, after which CFUs were counted. Bacterial survival is expressed relative to the untreated control.

### 4.7. Acid Survival Assay

*H. pylori* strains were grown in BB10 for 24 h, and the cells were suspended in buffer (20 mM Tris-HCl and 150 mM NaCl) at pH 7.0 or pH 2.0 at a concentration of ~2 × 10^7^ CFU/mL. The cell suspensions were incubated at 37 °C under microaerobic conditions for 5 and 10 min. The samples were serially diluted and spread onto CBA plates, and the CFUs were counted after 4 days of incubation under microaerobic conditions. The percentage of cell survival at pH 2.0 relative to that at pH 7.0 was calculated. The data are presented as relative survival to the wild-type.

### 4.8. Adhesion Assay

*H. pylori* wildtype or Δ*fur* mutant strains were suspended to homogeneity in RPMI 1640 to an optical density of 0.7, i.e., 10^8^ CFU/ml. AGS cells seeded in 24-well plates the day before were infected with the bacterial suspensions at an MOI of 100. After 2 h of incubation, the cells were washed three times with phosphate-buffered saline (PBS) to remove any unbound bacteria. Following the final wash, the cells were lysed with 1% saponin in RPMI 1640 for 5 min. The number of adhered CFU was determined by serial dilution and spreading the lysate on Columbia blood agar plates.

### 4.9. Statistical Analysis

GraphPad Prism 7 was used for all statistical analyses. Significant differences were determined by two-tailed and unpaired Student’s *t* tests or one-way analysis of variance (ANOVA) followed by Dunnett’s multiple-comparisons test. Differences with *p* values less than 0.05 were considered significant. Error bars represent the standard deviation.

## Figures and Tables

**Table 1:** Bacterial strains and plasmids used in this study.

Strain or Plasmid	Characteristics *^a^*	Source or Reference
**Strains**		
***E. coli* DH5α**		Invitrogen
*H. pylori* 67:21	Isolated from a patient with gastric ulcer	[37]
***H. pylori* 67:21** **Δ*arsR***	Cm^r^; *H. pylori* 67:21 *arsR* mutant	This study
***H. pylori* 67:21** **Δ*flgR***	Cm^r^; *H. pylori* 67:21 *flgR* mutant	This study
***H. pylori* 67:21** **Δ*fur***	Cm^r^; *H. pylori* 67:21 *fur* mutant	This study
*L. gasseri* Kx 110 A1	Isolated from human gastric biopsy specimen	[15]
*L. oris* Kx 112 A1	Isolated from human gastric biopsy specimen	[15]
**Plasmids**		
**pACYC184**	Cm^r^, Tet^r^; *E. coli* cloning vector	ATCC
**pJET1.2./blunt**	Amp^r^; *E. coli* cloning vector	ThermoFisher Scientific
**pJET-arsR-cmr**	pJET1.2./blunt derivative containing *arsR-Cmr* fusion PCR product	This study
**pJET-flgR-cmr**	pJET1.2./blunt derivative containing *flgR-Cmr* fusion PCR product	This study
**pJET-fur-cmr**	pJET1.2./blunt derivative containing *fur-Cmr* fusion PCR product	This study

*^a^* Tet^r^, tetracycline resistant; Cm^r^, chloramphenicol resistant; Amp^r^, ampicillin resistant.

**Table 2:** Primers used in this study.

Primers	Sequence (5′-3′) *^a^*	Purpose	Source
arsS_up_F	GGCATTAGTGCGGCTAACACACAAAAT	Cloning	This study
arsS_up_R	ACTGATTTAGTGTATGATGGAACCCCTTAACTCCTTATTAGAAT	Cloning	This study
arsS_down_F	ATAATAAGCGGATGAATGGCAGAAAAACAAAAAGAGAGAACATG	Cloning	This study
arsS_down_R	TTAGTGGAATAACTCATGATGGGCGTGT	Cloning	This study
arsS_Cmr_F	GGAGTTAAGGGGTTCCATCATACACTAAATCAGTAAGT	Cloning	This study
arsS_Cmr_R	CTTTTTGTTTTTCTGCCATTCATCCGCTTATTATCACT	Cloning	This study
flgR_up_F	TAGAAGATCAAGAATTTTTAATTTCGT	Cloning	This study
flgR_up_R	TTAGTGTATGATGGTCTTCTTCCTTTCTAAAAATATCT	Cloning	This study
flgR_down_F	ATAAGCGGATGAATGGCAATAAAGGCACGATCTTTTTAGAT	Cloning	This study
flgR_down_R	CCACGACGCCTAAAAGCTCTCGC	Cloning	This study
flgR_Cmr_F	AAAGGAAGAAGACCATCATACACTAAATCAGTAAGT	Cloning	This study
flgR_Cmr_R	CTTTATTGCCATTCATCCGCTTATTATCACTTAT	Cloning	This study
fur_up_F	CTACCCTGAAGCGCGCATCAT	Cloning	This study
fur_up_R	AGTGTATGATGGCCTTATCCGTAAAATGATTTTTATAACT	Cloning	This study
fur_down_F	ATGAATGGCAGAGTGAATGTTAAAAGATTTTAAAAAAG	Cloning	This study
fur_down_R	GAAAAGCTCTTTTGTGGAGTTTTTTG	Cloning	This study
fur_Cmr_F	ATTTTACGGATAAGGCCATCATACACTAAATCAGTAAGTTG	Cloning	This study
fur_Cmr_R	ATCTTTTAACATTCACTCTGCCATTCATCCGCTTATTAT	Cloning	This study

## Data Availability

The datasets used and/or analyzed during the current study are available from the corresponding author upon reasonable request.
